# Dr. Phineas G. C. Hunt

**Published:** 1896-06

**Authors:** 


### Obituary.

### DR. PHINEAS G. C. HUNT.

Dr. Hunt died at Indianapolis April 24, in his sixty-eighth year, of heart complications.

He was born in 1827, in Champaign County, Ohio. After passing through the preliminary training at school, he began the study of dentistry with his brother in Indianapolis. His practice extended over forty-five years in one community, a length of time in one place not often exceeded.

Dr. Hunt early discarded the narrow prejudices prevalent at the beginning of his career and took an active part in all the educational movements, which have since made dentistry worthy the name of a liberal profession. He introduced many valuable devices in dentistry and was a frequent contributor to dental periodicals.

The honorary degree of Doctor of Dental Surgery was conferred upon him by the Ohio College of Dental Surgery in 1870. The honorary degree of Doctor of Medicine had been previously conferred by the Indiana Medical College.

He was elected president of the American Dental Association in 1872, and was also elected president of the Indiana State Association in 1861, and was made presiding officer of the Indiana State Board of Examiners, which position he continuously held.

He was one of the trustees of the Indiana Dental College, in which his son, Dr. George E. Hunt, is professor of Dental Pathology and Therapeutics.

Dr. Hunt was a thirty-third degree Mason and had held high offices in that body.

Of four brothers, three of whom were dentists, the subject of this sketch was the last to die, and in his departure the profession of Indiana and the country at large will miss an earnest, liberal worker, unfailing in his efforts to carry his chosen profession to a broader conception of its duties.

